# Racial and Ethnic Disparities in Co-Occurrence of Nocturnal Hypertension and Nocturnal Blood Pressure Decreases

**DOI:** 10.1001/jamanetworkopen.2023.52227

**Published:** 2024-01-18

**Authors:** Neil Zhang, Tzu Yu Huang, Susan Cheng, Joseph E. Ebinger

**Affiliations:** 1 Smidt Heart Institute, Department of Cardiology, Cedars-Sinai Medical Center, Los Angeles, California

## Abstract

This cross-sectional study assesses racial and ethnic disparities in co-occurrence of nocturnal hypertension and blunted nocturnal decreases in blood pressure.

## Introduction

Racial and ethnic disparities persist in blood pressure (BP) phenotypes. The co-occurrence of nocturnal hypertension (NH) and blunted nocturnal “dipping” (nondipping) BP represents a higher-risk condition^1^ that remains understudied and underrecognized. Because NH has been associated with circadian disturbance and nondipping BP with neurohormonal alterations, we hypothesized that their co-occurrence is more prevalent among groups with higher rates of NH and nondipping BP assessed separately, particularly among Black patients, given that social determinants of health (SDoH) and medical disparities can affect mechanisms underlying both phenotypes.^2^

## Methods

In this cross-sectional study, we reviewed results from automated BP monitoring (ABPM) between January 1, 2013, and December 31, 2022, at a large urban academic medical center; results with incomplete BP data were excluded. Demographics and comorbidities (diabetes, coronary artery disease, myocardial infarction, heart failure, kidney disease, and hypertension) were obtained from the electronic health record (EHR), the latter via *ICD-10* codes (eTable in Supplement 1). Race and ethnicity collection for the EHR, categorized based on US Office of Management and Budget standards, was through self-report or staff queries, although patient-level details were unavailable.^3^ We used guideline-based definitions of NH (mean evening systolic BP >110 mm Hg) and nondipping BP (<10% decrease in daytime-to-nighttime mean systolic BP or increase in daytime-to-nighttime mean BP). Patients were categorized as having NH, nondipping BP, co-occurrent NH and nondipping BP, or normal nocturnal BP. Cedars-Sinai Medical Center institutional review board approved this study and waived patient consent because the study used deidentified retrospective data.

We used multivariable-adjusted logistic regression to identify characteristics associated with co-occurrent NH and nondipping BP and tested for interaction between covariates. Asian patients were the reference group because they demonstrated the lowest adjusted outcome rate, allowing for concordant directionality of associations across groups. This study followed the STROBE reporting guideline.^4^ Analysis was performed with R, version 4.2.3. *P* values were 2-sided and deemed statistically significant at *P* < .05.

## Results

A total of 1619 patients completed ABPM, with 34 excluded for incomplete data. Of the remaining 1585 patients (median age, 57 years [IQR, 40-72 years]), 46.4% were male; 5.6% Asian, 12.3% Hispanic or Latinx, 7.9% non-Hispanic Black, and 67.1% non-Hispanic White; 70.5% had hypertension; and 41.3% had co-occurrent NH and nondipping BP (Table). In unadjusted analyses, prevalence of NH, nondipping BP, and co-occurrent NH and nondipping BP was highest among men, non-Hispanic Black patients, and individuals with kidney disease. After adjustment, male sex (odds ratio [OR], 1.73; 95% CI, 1.33-2.25), increasing age (OR, 1.17; 95% CI, 1.09-1.26 per decade), kidney disease (OR, 2.19; 95% CI, 1.34-3.65), and hypertension (OR, 1.81; 95% CI, 1.31-2.51) were associated with co-occurrent NH and nondipping BP (Figure). Non-Hispanic Black patients were more likely to have co-occurrent NH and nondipping BP (OR, 2.67; 95% CI, 1.32-5.51). Significant interactions were found between male sex and hypertension diagnosis.

**Table.** Patient Characteristics

Characteristic	Patient group				
	Total^a^	Normal nocturnal BP and nondipping BP	Nocturnal hypertension	Nocturnal nondipping BP	Nocturnal hypertension and nondipping BP
No. (%)	1585 (100)	393 (24.8)	978 (61.7)	869 (54.8)	655 (41.3)
Male, No. (%)	735 (46.4)	136 (18.5)	546 (74.3)	416 (56.6)	363 (49.4)
Daytime systolic BP, mean (SD), mm Hg	126.9 (15.9)	118.3 (10.4)	134.7 (13.0)	125.9 (16.5)	132.3 (13.2)
Daytime diastolic BP, mean (SD), mm Hg	75.6 (10.2)	73.6 (8.5)	78.3 (10.3)	74.4 (10.1)	76.7 (9.9)
Race and ethnicity, No. (%)					
Asian	89 (5.6)	20 (22.5)	57 (64.0)	45 (50.6)	33 (37.1)
Hispanic or Latinx	195 (12.3)	52 (26.7)	122 (62.6)	103 (52.8)	82 (42.1)
Non-Hispanic Black	125 (7.9)	17 (13.6)	98 (78.4)	80 (64.0)	70 (56.0)
Non-Hispanic White	1064 (67.1)	278 (26.1)	628 (59.0)	581 (54.6)	423 (39.8)
Other^b^	67 (4.2)	16 (23.9)	44 (65.7)	38 (56.7)	31 (46.3)
Unreported race and ethnicity	45 (2.8)	10 (22.2)	29 (64.4)	22 (48.9)	16 (35.6)
Comorbidities, No. (%)					
Diabetes	137 (8.6)	19 (13.9)	106 (77.4)	91 (66.4)	79 (57.7)
Coronary artery disease	291 (18.4)	48 (16.5)	212 (72.9)	187 (64.3)	156 (53.6)
Myocardial infarction	34 (2.1)	7 (20.6)	23 (67.6)	22 (64.7)	18 (52.9)
Heart failure	127 (8.0)	18 (14.2)	88 (69.3)	90 (70.9)	69 (54.3)
Hypertension	1118 (70.5)	238 (21.3)	801 (71.6)	615 (55.0)	536 (47.9)
Kidney disease	131 (8.3)	12 (9.2)	107 (81.7)	98 (74.8)	86 (65.6)

Abbreviation: BP, blood pressure.

^a^ Percentages in the “Total” column represent the percentages of the total population. Percentages in all the remaining rows are calculated across rows.

^b^ Includes American Indian or Alaska Native and Native Hawaiian or Other Pacific Islander.

**Figure.**
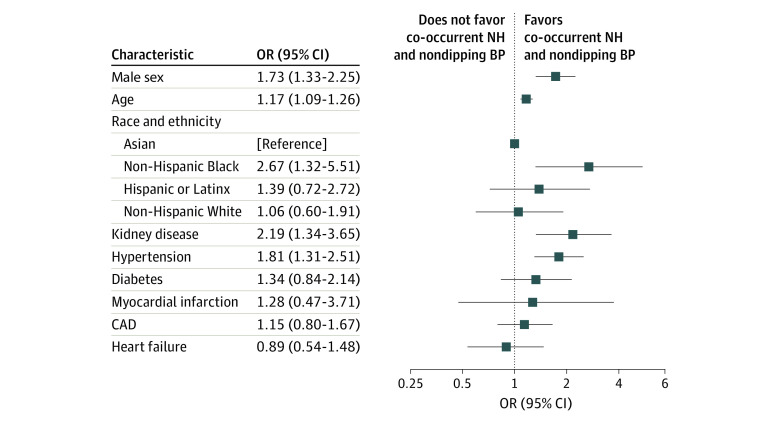
Association of Demographic and Clinical Factors With Co-Occurrent Nocturnal Hypertension and Nondipping Blood Pressure Status Estimates are derived from multivariate-adjusted models. CAD indicates coronary artery disease, NH, nocturnal hypertension; OR, odds ratio.

## Discussion

Co-occurrent NH and nondipping BP was prevalent among 41.3% of patients receiving ABPM, particularly men, individuals with kidney disease, and non-Hispanic Black patients. Prior studies have observed that either NH or nondipping BP is more prevalent among Black populations^5^; our study clarifies that these conditions tend to co-occur among this population.

Mechanisms have been proposed to explain racial and ethnic variations in NH and nondipping BP, including diet and comorbidities, although these disparities may be predominantly associated with SDoH. Nocturnal hypertension and nondipping BP are more closely associated with cardiovascular events than daytime BP, which may place Black patients at even greater cardiovascular risk than appreciated by standard BP assessments.^6^

Several limitations merit consideration, including the low proportion of non-Hispanic Black patients, although the numerical population is large. Datasets derived from EHRs carry limitations, including nonstandardized definitions (eg, race and ethnicity). Participant-level SDoH and medication data were not available. Our findings indicate the need for prospective studies to confirm the prevalence and correlates of co-occurrent NH and nondipping BP and to examine their synergistic roles contributing to or augmenting disparities in cardiovascular risk.

## Notes

Supplement 1. **eTable.** *ICD-10* Codes Used for Identification of Clinical Comorbidities

Supplement 2.
Data Sharing Statement
