# Supplementary material for: Racial and Ethnic Disparities in Co-Occurrence of Nocturnal Hypertension and Nocturnal Blood Pressure Decreases
Source: JAMA Netw Open. 2024 Jan 18;7(1):e2352227. doi: 10.1001/jamanetworkopen.2023.52227 (PMC10797446; doi:10.1001/jamanetworkopen.2023.52227)
Supplement: Supplement 1. — eTable. ICD-10 Codes Used for Identification of Clinical Comorbidities [file jamanetwopen-e2352227-s001.pdf]

Supplementary Online Content

Zhang N, Huang TY, Cheng S, Ebinger JE. Racial and ethnic disparities in co-occurrence of nocturnal hypertension and nocturnal blood pressure decreases. *JAMA Netw Open.* 2024;7(1):e2352227.
doi:10.1001/jamanetworkopen.2023.52227

eTable. *ICD-10* Codes Used for Identification of Clinical Comorbidities

This supplementary material has been provided by the authors to give readers additional information about their work.

© 2024 Zhang N et al. *JAMA Network Open.*

eTable. *ICD-10* Codes Used for Identification of Clinical Comorbidities

Comorbidity	ICD-10 Codes
Diabetes Mellitus	E10.*, E11.*, E12.*, E13.*, E14.*
Coronary Artery Disease	I25.4, I25.1*, I25.7*, I25.8*
Myocardial Infarction	I25.2, I25.6, I22.0, I21.0, I21.*, I22.*
Heart Failure	I11.0, I13.0, I13.2, I25.5, I42.0, I42.5, I42.6, I42.7, I42.8, I42.9, I50.*, I43.*
Hypertension	I10*', I11*, I12*, I13*, I15*, I16*
Renal Disease	I12.0, I12.9, I13.0, I13.1, I13.10, I13.11, I13.2, I13.9, N25.0, Z49.0, Z49.1, Z49.2, Z94.0, Z99.2, N03*, N04*, N05*, N18*, N19*

© 2024 Zhang N et al. *JAMA Network Open.*
