# Supplementary material for: Racial and Ethnic Disparities in Co-Occurrence of Nocturnal Hypertension and Nocturnal Blood Pressure Decreases
Source: JAMA Netw Open. 2024 Jan 18;7(1):e2352227. doi: 10.1001/jamanetworkopen.2023.52227 (PMC10797446; doi:10.1001/jamanetworkopen.2023.52227)
Supplement: Supplement 2. — Data Sharing Statement [file jamanetwopen-e2352227-s002.pdf]

# Data Sharing Statement

Zhang. Racial and Ethnic Disparities in Co-Occurrence of Nocturnal Hypertension and Nocturnal Blood Pressure Decreases. *JAMA Netw Open*. Published January 18, 2024. doi:10.1001/jamanetworkopen.2023.52227

**Data**
**Data available:** Yes
**Data types:** Deidentified participant data
**How to access data:** Requests for de-identified data may be directed to biodatacore@cshs.org and will be reviewed by the Office of Research Administration at Cedars-Sinai Medical Center prior to issuance of data sharing agreements, which are designed to ensure patient and participant confidentiality.
**When available:** With publication

**Supporting Documents**
**Document types:** None

**Additional Information**
**Who can access the data:** Upon reasonable request, de-identified data will be available to researchers whose proposed use of the data has been approved and following issuance of relevant institutional data sharing agreements.
**Types of analyses:** Upon reasonable request, de-identified data will be available for the purposes of research outlined in an approved proposal and following issuance of relevant institutional data sharing agreements.
**Mechanisms of data availability:** Upon reasonable request, de-identified data will be available to researchers following approval of a proposal and following issuance of a signed institutional data sharing agreement.
